# *Candida albicans* V132 induces trained immunity and enhances the responses triggered by the polybacterial vaccine MV140 for genitourinary tract infections

**DOI:** 10.3389/fimmu.2022.1066383

**Published:** 2022-11-24

**Authors:** Leticia Martín-Cruz, Alba Angelina, Ilayda Baydemir, Özlem Bulut, José Luis Subiza, Mihai G. Netea, Jorge Domínguez-Andrés, Oscar Palomares

**Affiliations:** ^1^ Department of Biochemistry and Molecular Biology, School of Chemistry, Complutense University of Madrid, Madrid, Spain; ^2^ Department of Internal Medicine and Radboud Center for Infectious Diseases, Radboud University Medical Centre, Nijmegen, Netherlands; ^3^ Inmunotek, Alcalá de Henares, Madrid, Spain; ^4^ Department of Immunology and Metabolism, Life and Medical Sciences Institute, University of Bonn, Bonn, Germany

**Keywords:** trained immunity, genito urinary infections, candida albicans V132, polybacterial preparation MV140, metabolic and epigenetic reprogramming

## Abstract

**Introduction:**

Recurrent urinary tract infections (RUTIs) and recurrent vulvovaginal candidiasis (RVVCs) represent major healthcare problems all over the world. Antibiotics and antifungals are widely used for such infectious diseases, which is linked with microbial resistances and microbiota deleterious effects. The development of novel approaches for genitourinary tract infections (GUTIs) such as trained immunity-based vaccines (TIbV) is therefore highly required. MV140 is a sublingual whole-cell heat-inactivated polybacterial preparation with demonstrated clinical efficacy for RUTIs. The sublingual heat-inactivated *Candida albicans* vaccine V132 has been developed for RVVCs. We previously showed that the combination of MV140 and V132 promotes potent Th1/Th17 and regulatory T-cell responses against antigens contained in the formulation and unrelated antigens. The specific contribution of each preparation to such effects and the underlying molecular mechanisms remain incompletely understood.

**Methods:**

PBMC and monocytes were isolated from healthy donors and *in vitro* stimulated with V132, MV140 or MV140/V132. After 6 days of resting, cells were reestimulated with LPS and MV140. Analysis of cytokine production by ELISA, Seahorse assays for functional metabolic experiments and chromatin immunoprecipitation assays were performed. BALB/c mice were intraperitoneally and sublingually immunized with V132.

**Results:**

We uncover that V132 induces trained immunity in human PBMCs and purified monocytes, significantly increasing the responses triggered by subsequent stimulation with MV140. Mechanistically, V132 drives metabolic rewiring towards increased glycolysis and oxidative phosphorylation and induces epigenetic reprogramming that enhances the transcription of the pro-inflammatory genes *IL6* and *TNFA*. Splenocytes and peritoneal cells from V132-immunize mice show increased responses upon *in vitro* stimulation with MV140. Remarkably, splenocytes from sublingually V132-immunized and MV140 *in vivo* treatment mice show stronger Th17 responses than mice exposed to excipients upon *in vitro* stimulation with MV140.

**Conclusion:**

Overall, we provide novel mechanistic insights into how V132-induced trained immunity enhances both innate and adaptive immune responses triggered by MV140, which might open the door for new interventions for GUTIs with important clinical implications.

## Introduction

Urinary tract infections (UTIs) constitute one of the most prevalent bacterial infections, representing a major healthcare problem of increasing prevalence worldwide, especially in women (1). Despite antibiotic treatments, more than 30% of women experience recurrent UTIs (RUTIs), defined as more than three infections within 12 months (2, 3). Long-term treatment with antibiotics might favor the growth of drug-resistant microorganisms as well as microbiota alterations in the gastrointestinal tract and vagina (4, 5). These alterations facilitate the aberrant proliferation of yeasts such as *Candida albicans*, which is associated with vulvovaginal candidiasis (VVCs) (6). In this regard, around 75% of women experience one episode of VVC during their lifetime and up to 5% develop recurrent episodes (RVVCs) (7–10). Topical and oral antifungal formulations are the standard prescribed treatments (11, 12), hence repeated and prolonged antifungal therapy also increases the risk to develop resistances of the pathogens to the available medication (13, 14).

Mucosal bacterial and fungal vaccines have been suggested as promising alternative strategies for genitourinary tract infections (GUTIs) (15–18). Some of these preparations containing live-attenuated or inactivated pathogens are trained immunity-based vaccines (TIbV), which might confer broad protection against target infections while also exhibiting heterologous effects (19–24). Trained immunity is the process by which a primary stimulus reprogram the function of innate immune cells favoring an augmented response against an either related or unrelated secondary challenge (25, 26). Induction of trained immunity was initially described in monocytes, macrophages and NK cells, but other cell types such as neutrophils, dendritic cells (DCs), innate lymphoid cells and bone marrow progenitors can also display a trained immunity phenotype (27).

Metabolic and epigenetic reprograming are the hallmarks of trained immunity. Metabolism play a major role in the induction and preservation of trained immunity (28, 29). In particular, activation of trained cells is connected with different metabolic pathways connecting the energetic need and the functional activity. An increased glycolysis and oxidative phosphorylation with a higher glucose consumption and production of lactate and ATP are the main cellular metabolic pathways involved in trained immunity (30–33). Several metabolites are fundamental to activate and modulate the epigenetic remodeling in trained cells (29). To facilitate an enhanced responsiveness upon a secondary stimulus, trained cells modify chromatin structure and accessibility of promoters of genes codifying for pro-inflammatory cytokines such as TNFα or IL-6, and glycolytic enzymes (29, 32).

MV140 (Uromune) is a whole-cell, heat-inactivated, sublingual bacterial preparation containing equal proportions of those bacteria causing most of RUTIs across Europe (*Escherichia coli*, *Proteus vulgaris*, *Klebsiella pneumoniae* and *Enterococcus faecalis*) (21). This sublingual vaccine significantly reduces the infection rates in patients suffering from RUTIs (34–41). In addition, MV140 induces a potent activation of human DCs and the generation of Th1, Th17 and regulatory T (Treg) cells (21, 22). On the other hand, V132 is a whole-cell, heat-inactivated, *C. albicans* sublingual vaccine that has been designed as a novel therapy for RVVCs (22). The combination of MV140 with V132 in a single preparation primes human DCs to induce potent Th1/Th17 and Treg cells (22). Sublingual administration of MV140/V132 enhances proliferative responses of mice splenic CD4^+^ T cells against related and unrelated antigens, suggesting trained immunity mechanisms (22). The specific contribution of each preparation to these effects and the underlying molecular mechanisms remains unknown. Herein, we show for the first time that sublingual vaccine *C. albicans* V132 induces trained immunity and enhances responses to MV140 both *in vitro* and *in vivo*. V132 induces metabolic and epigenetic rewiring characterized by a shift of cellular metabolism towards aerobic glycolysis and oxidative phosphorylation, enhancing transcription of pro-inflammatory cytokines. *In vivo*, sublingual administration of V132 in mice potentiates IL-17-mediated responses after *in vitro* stimulation with MV140. Our findings shed light into novel molecular mechanisms involved in the beneficial effects of these microbial sublingual preparations for GUTIs, which might well contribute to open new interventions with multiple clinical implications.

## Materials and methods

### Media and reagents

RPMI culture medium Dutch modified (Invitrogen) supplemented with 50 μg/mL gentamicin, 2 mM Glutamax (Gibco), and 1 mM pyruvate (Gibco) was used for human *in vitro* experiments. RPMI 1640 medium (Lonza) supplemented (cRPMI) with 10 % heat‐inactivated fetal bovine serum (Gibco), 100 μg/mL normocin (*In vivo* Gen), 50 μg/mL penicillin-streptomycin, 1% nonessential amino acids, 1% MEM vitamins and 1 mM sodium pyruvate (all from Life Technologies) was used for animal experiments. V132 composed of whole heat-inactivated *C. albicans*, MV140 (Uromune) composed of whole heat-inactivated bacteria (25% *E. coli*, 25% *P. vulgaris*, 25% *K. pneumoniae* and 25% *E. faecalis*), MV140/V132 (combination of MV140 and V132) and control excipients (glycerol, saline, pineapple flavor), identical for MV140 or V132, were provided by Inmunotek S.L. Lipopolysaccharide (LPS) from *E. coli* O55:B5 (Sigma-Aldrich) was used.

### PBMC and monocyte isolation

Buffy coats from healthy donors were obtained after written informed consent (Sanquin Blood Bank, Nijmegen, the Netherlands). Peripheral blood mononuclear cells (PBMC) isolation was performed by using Ficoll-Paque density gradient media (GE Healthcare). Cells were washed twice in PBS, re-suspended in RPMI culture medium and counted. Monocyte isolation was performed by hyper-osmotic Percoll (Sigma) density gradient centrifugation (580*g*, 15 min). The interphase layer was collected, and cells were washed with cold PBS, re-suspended in RPMI culture medium Dutch modified and counted. An extra purification step was added by adhering Percoll-isolated monocytes or Ficoll-isolated PBMC to a flat-bottom 96-well plates for 1 h at 37°C. Subsequently, monocytes were washed with warm PBS to yield maximal purity.

### *In vitro* trained immunity experiments

Trained immunity experiments were performed as previously described (42). 100 μL monocytes at 1 x 10^6^ cells/mL or PBMC at 5 x 10^6^ cells/mL density were added to a flat-bottom 96-well plates. Cells were incubated with control excipients (containing all excipients except the microbial components), V132 (3 FTU, Formazine Turbidity Units, per mL, as turbidity measurement for the monitoring of fungal suspensions), MV140 (10^7^ bact. per mL) or MV140/V132 (MV140, 10^7^ bact. per mL, and V132, 3 FTU per mL, as turbidity measurement for the monitoring of fungal suspensions) for 24 h. Supernatants from monocytes were collected 24 h after stimulation. Cells were washed once with 200 μL of warm PBS and incubated for 6 days in RPMI culture medium Dutch modified with 10% human pooled serum. On day 6, supernatants were collected for lactate measurement. Cells were re-stimulated for 24 h with 10 ng/mL LPS or 10^7^ bact. per mL MV140 to assess the responsiveness of the cells to heterologous secondary stimulation, after which supernatants were collected.

### Cytokine quantification

Cytokine production was quantified in supernatants using commercial ELISA kits for human IL-6, TNFα, IL-1β, IL-10, IL-1RA (R&D Systems); mouse IL-6, IFNγ, (BD Biosciences); TNFα, IL-10 (Invitrogen); and IL-17A (R&D Systems), following manufacturer’s instructions.

### Lactate measurement

Lactate concentration was measured from supernatants using a Lactate Fluorometric Assay Kit. Briefly, lactate was oxidized resulting in H_2_O_2_ which was coupled to the conversion of Amplex Red reagent to fluorescent resorufin by horseradish peroxidase (HRP).

### Viability assays

Cell viability was analyzed using CytoTox 96 Non-Radioactive Cytotoxicity Assay (Promega), following manufacturer’s recommendations. Briefly, released lactate dehydrogenase (LDH) upon cell lysis is measured with a 30-minute coupled enzymatic assay, which results in conversion of a tetrazolium salt (INT) into a red formazan product. The amount of color formed is proportional to the number of lysed cells.

### Metabolic analysis

Adherent monocytes were obtained by incubating 5 x 10^7^ PBMC in 10 cm Petri dishes for 1 h. The adherent monocytes were treated with 10 mL of RPMI culture medium Dutch modified containing control excipients, V132, MV140 or MV140/V132 for 24 h, washed once with warm PBS and incubated in RPMI culture medium Dutch modified with 10% human pooled serum at 37°C, 5% CO_2_. Following 6 days in culture, cells were detached with Versene solution (ThermoFisher Scientific) and 5 x 10^5^ cells were plated to overnight-calibrated cartridges in DMEM medium supplemented with 1 mM glutamine (pH adjusted to 7.4) and incubated for 1 h in a non-CO_2_-corrected incubator at 37°C. Extracellular acidification rate (ECAR) and oxygen consumption rate (OCR) in response to 10 mM glucose, 1 μM oligomycin, 1 μM carbonyl cyanide 4-(trifluoromethoxy) phenylhydrazone (FCCP), and 1.25 μM rotenone and 1.5 μM antimycin A injections were measured using a Seahorse XFe96 Analyzer (Agilent). A complete ECAR study was performed in two consecutive stages: basal extracellular acidification (after glucose injection), and mitochondrial complex V inhibition (oligomycin). A complete OCR study was performed in four consecutive stages: basal respiration (without drugs), mitochondrial complex V inhibition (oligomycin), maximal respiration induction (FCCP), and electron transportation chain inhibition (rotenone and antimycin A).

### Chromatin immunoprecipitation

Adherent monocytes were obtained by incubating 5 x 10^7^ PBMC in 10 cm Petri dishes for 1 h. The adherent monocytes were treated with 10 mL of RPMI culture medium Dutch modified containing control excipients, V132, MV140 or MV140/V132 for 24 h, washed once with warm PBS and incubated in RPMI culture medium Dutch modified with 10% human pooled serum at 37°C, 5% CO_2_. Following 6 days in culture, cells were detached with Versene solution (ThermoFisher Scientific) and fixed in 1% methanol-free formaldehyde. Afterward, cells were sonicated using a Diagenode Bioruptor UCD-300 for 10 min (30 seconds on; 30 seconds off). 33 μL of the solution containing chromatin was incubated with 255 μL of dilution buffer, 12 μL of protease inhibitor cocktail and 1 μg of H3K27ac or H3K9me3 antibodies (Diagenode), overnight at 4°C with rotation. Protein A/G magnetic beads were washed in dilution buffer with 0.15% SDS and 0.1% BSA, added to the chromatin/antibody mix and rotated for 60 min at 4°C. Beads were washed with 500 μL buffer for 5 min at 4°C with five rounds of washes. After washing chromatin was eluted using elution buffer for 20 min. Supernatant was collected, 8 μL 5 M NaCl and 1 μL proteinase K were added, and samples were de-crosslinked at 64°C for 4 h. DNA was isolated with the MinElute PCR Purification Kit (QIAGEN), following manufacturer’s protocol. Real-time quantitative PCR was performed using the SYBR Green method. Samples were analyzed by a comparative Ct method according to the manufacturer’s instructions. The sequences of the used pair primers were: *IL6-1* (forward, TCGTGCATGACTTCAGCTTT; reverse GCGCTAAGAAGCAGAACCAC), *IL6-2* (forward, AGGGAGAGCCAGAACACAGA; reverse GAGTTTCCTCTGACTCCATCG), *TNFA1* (forward, AGAGGACCAGCTAAGAGGGA; reverse AGCTTGTCAGGGGATGTGG), and *TNFA2* (forward, GTGCTTGTTCCTCAGCCTCT; reverse ATCACTCCAAAGTGCAGCAG).

### Mice experiments

All mice procedures included in this study were reviewed and ethically approved by Universidad Complutense de Madrid (UCM) and Comunidad Autónoma de Madrid (CAM) within the context of project SAF-2017-84978-R, (CAM: ref.10/250312.9/18). BALB/c mice (female, 6 weeks old, Charles River) were intraperitoneally immunized with 20 μL of control excipients or V132 (300 FTU per mL) at day 0 and 3 and sacrificed 4 days after the last immunization. In another animal model, BALB/c mice (6 weeks old) were sublingually immunized for 5 consecutive days during 2 weeks with 20 μL of control excipients or V132 (300 FTU per mL) and sacrificed 3 days after last immunization. Moreover, some immunized mice were sublingually administered with 20 μL of MV140 (10^9^ bact. per mL) 3 days after last sensitization and sacrificed after 8 days of the MV140 administration. Peritoneal cells and spleens were collected.

### Peritoneal lavage and peritoneal cell culture

Peritoneal cells were collected with PBS/EDTA 2mM and resuspended in cRPMI. Duplicates of 0.25 x 10^6^ peritoneal cells were cultured *in vitro* with control excipients or MV140 (10^6^ bact. per mL) in flat-bottom 96-well plates for 24 h at 37°C and 5% CO_2_. Cell-free supernatants of duplicates were pooled and used to quantify IL-6 and TNFα.

### Spleen processing and splenocyte cell culture

Spleens were minced and filtered through 40 μm nylon cell strainers to obtain a single-cell suspension. Then, red blood cells were lysed with ACK lysis buffer before being resuspended in cRPMI. Splenocyte viability was assessed using Trypan Blue exclusion. Triplicates of 0.8 x 10^6^ splenocytes were cultured *in vitro* with control excipients or MV140 (10^5^-10^7^ bact. per mL) in flat-bottom 96-well plates. After 24 or 72 h of culture at 37°C and 5% CO_2_, the triplicates were pooled and cell-free supernatants were used to quantify IL-6, TNFα, IL-17, IFNγ and IL-10 by ELISA.

### Statistics

All the data are expressed as means ± s.e.m. of the indicated parameters. One-way ANOVA, Wilcoxon test or Unpaired Student *t t*est for statistical analysis were performed using GraphPad Prism software, version 6.0. Significance was defined as *P < 0.05, **P < 0.01, ***P < 0.001, and ****P < 0.0001.

## Results

### Exposure of human peripheral blood mononuclear cells to V132 enhances pro-inflammatory cytokine production after secondary stimulation with LPS

**Figure 1:**
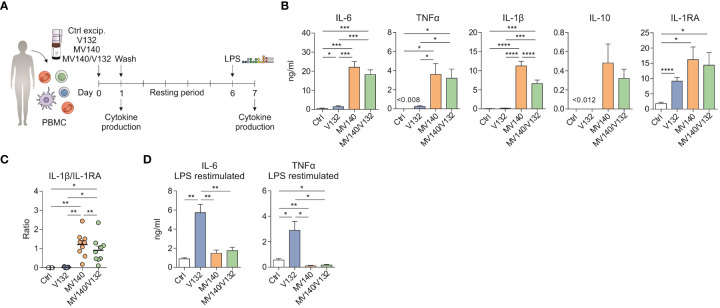
V132 induces trained immunity in human PBMC. **(A)** Human *in vitro* model for the induction of trained immunity in human PBMC. **(B)** Cytokine production after stimulation of human PMBC with control excipients (ctrl), V132, MV140 or MV140/V132 for 24 h in cell-free supernatants (n = 6-11 donors of two or four independent experiments). **(C)** IL-1β/IL-1RA ratio produced by human PBMC after 24 h in the indicated conditions (n = 9 donors of four independent experiments). **(D)** PBMC were incubated for 24 h with ctrl, V132, MV140 or MV140/V132. After 6 days, they were re-stimulated with LPS and cytokines were measured 24 h later (n = 11 donors of four independent experiments). Values are mean ± SEM. Statistical significance was determined using One-way ANOVA. *P < 0.05, **P < 0.01, ***P < 0.001, and ****P < 0.0001.

To assess whether priming of human peripheral blood mononuclear cells (PBMC) with V132, MV140, or a simultaneous combination of V132 and MV140 would increase PBMC’s responses upon second stimulation, an *in vitro* model for the induction of trained immunity was employed (43, 44). As shown in **Figure 1A**, PBMC were initially treated with control excipients (ctrl), V132 alone, MV140 alone, and the combination of MV140 and V132 (MV140/V132) and the cytokine signature after 24 h of stimulation quantified. Next, cells were washed, left resting for 6 days with medium, and then re-stimulated with LPS to assess cytokine production after 24 h (**Figure 1A**). PBMC stimulation with V132 alone induced significant IL-6 and anti-inflammatory IL-1RA production (**Figure 1B**). Exposure to MV140 and MV140/V132 significantly increased the production of IL-6, TNFα and IL-1β compared to control excipients or V132 (**Figure 1B**), without affecting cell viability (**Supplementary Figure 1A**). PBMC stimulated with MV140 alone produced significantly increased IL-1β compared to the MV140/V132 combination (**Figure 1B**). Although overall IL-10 production was higher after stimulation with MV140 than with control, V132 or MV140/V132, significant differences were not detected (**Figure 1B**). MV140 or MV140/V132-stimulated PBMC also significantly produced higher IL-1RA levels after 24 h, with higher levels observed after MV140 or MV140/V132 than V132 stimulation (**Figure 1B**). Consequently, the IL-1β/IL-1RA ratio was significantly higher when PBMC were stimulated with MV140 or MV140/V132 than control excipients or V132, and slight but higher with MV140 than with MV140/V132 (**Figure 1C**). PBMC conditioned with V132 displayed a trained response on day 7 after re-stimulation with LPS resulting in a significant IL-6 and TNFα production than control excipients (**Figure 1D**). This was not the case when PBMC were stimulated with MV140, and remarkably not with V132 in the presence of MV140 (MV140/V132) (**Figure 1D**). No IL-10 production was observed on day 7 in these cell cultures (data not shown). Collectively, these results suggest that in the assayed conditions, V132 but not MV140 or MV140/V132 induced trained immunity in human PBMC (**Figure 1D**).

### Exposure of human PBMC to V132 induces metabolic and epigenetic reprogramming

**Figure 2:**
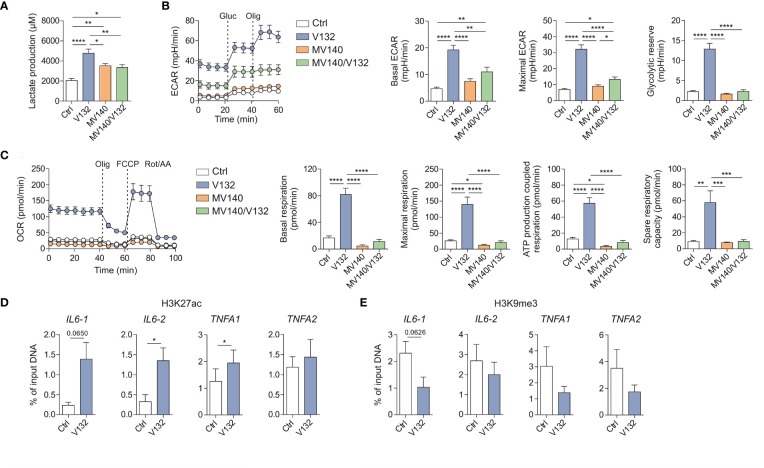
V132-trained cells exhibit metabolic and epigenetic rewiring. Human PBMC were incubated for 24 h with control excipients (ctrl), V132, MV140 or MV140/V132. On day 6 (prior to restimulation), metabolic and epigenetic assays were performed. **(A)** Lactate production by human PBMC was quantified (n = 12 donors of four independent experiments). **(B)** Kinetic study of extracellular acidification rate (ECAR) in ctrl-, V132-, MV140-, or MV140/V132-trained PBMC by sequential addition of glucose (Gluc) and oligomycin (Olig) (n = 7 donors of three independent experiments, 5 repeated measures of each donor). **(C)** Kinetic study of mitochondrial oxygen consumption rate (OCR) in ctrl-, V132-, MV140-, or MV140/V132-trained PBMC by sequential addition of oligomycin (Olig), (FCCP) and rotenone/antimycin A (Rot/AA) (n = 7 donors of three independent experiments, 5 repeated measures of each donor). H3K27ac **(D)** and H3K9me3 **(E)** histone modifications were determined at the promoter sites of *IL6* and *TNFA* using two different primers for each one (n = 5-6 donors of two independent experiments). Values are mean ± SEM. Statistical significance was determined using One-way ANOVA **(A-C)** or Paired Student *t* test **(D, E)**. *P < 0.05, **P < 0.01, ***P < 0.001, and ****P < 0.0001.

Trained cells undergo strong metabolic and epigenetic reprogramming, two major molecular mechanisms underlying trained immunity (26). Therefore, we first studied the metabolic changes imprinted by V132 in PBMCs compared to those induced by control excipients, MV140 or MV140/V132. PBMC treated with V132, MV140 or MV140/V132 produced significantly higher lactate concentrations in cell culture supernatants after 6 days of resting (prior to re-stimulation) compared to control cultures with excipients, reflecting an increase in the activity of glycolysis (**Figure 2A**). Remarkably, the levels of lactate produced by V132-trained PBMC were significantly higher than MV140- or MV140/V132-treated cells (**Figure 2A**). To further verify these results, we performed functional metabolic experiments using a Seahorse bioanalyzer to monitor real-time extracellular acidification rate (ECAR) and mitochondrial oxygen consumption rate (OCR) after 6 days of resting of the initially exposed PBMC. V132-trained PBMC showed an increased basal and maximal ECAR, as well as glycolytic reserve compared to PBMC stimulated with MV140, MV140/V132 or control excipients (**Figure 2B**). Only a slight but significant increase in basal and maximal ECAR was observed after the treatment with MV140/V132 compared to MV140 or control excipients (**Figure 2B**). In addition, basal and maximal respiration, ATP production coupled to respiration, and spare respiratory capacity were significantly higher after V132 training than the other assayed stimuli, also supporting the induction of mitochondrial oxidative phosphorylation in V132-trained PBMC (**Figure 2C**).

To determine whether the enhanced production of pro-inflammatory cytokines after the second re-stimulation with LPS might be linked to V132-induced epigenetic changes, we studied chromatin status near the *IL6* and *TNFA* genes by quantifying H3K27ac and H3K9me3 histone marks by chromatin immunoprecipitation (ChIP) analysis. H3K27ac is previously described as the histone mark associated with active chromatin and increased gene transcription in trained immunity (45). In contrast, H3K9me3 is considered a repressor mark (46). V132-trained PBMC display an increase of H3K27ac and a decrease of H3K9me3 at the promoters of *IL6* and *TNFA* (**Figure 2D, E**, respectively). Collectively, these results demonstrate that the induction of trained immunity with V132 in PBMC is mediated by metabolic and epigenetic reprogramming.

### V132 induces trained immunity in purified human monocytes

**Figure 3:**
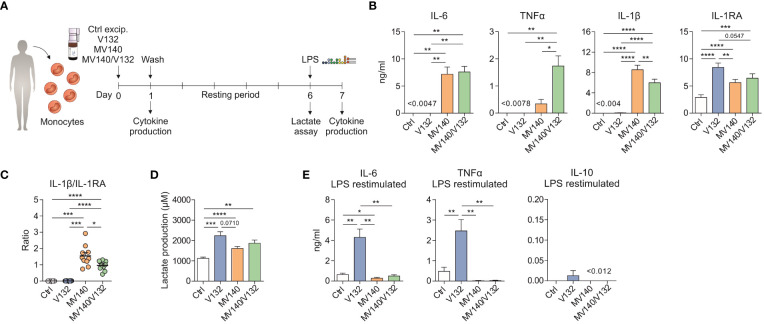
V132 induces trained immunity in human monocytes. **(A)** Human *in vitro* model for the induction of trained immunity in human monocytes. **(B)** Cytokine production after stimulation of human monocytes with control excipients (ctrl), V132, MV140 or MV140/V132 for 24 h in cell-free supernatants (n = 10 donors of four independent experiments). **(C)** IL-1β/IL-1RA ratio produced by human monocytes after 24 h in the indicated conditions (n = 10 donors of four independent experiments). **(D)** Lactate production by human monocytes 6 days after 24 h stimulation with ctrl, V132, MV140 or MV140/V132, followed by rest in culture media (n = 11 donors of four independent experiments). **(E)** Monocytes were incubated for 24 h with ctrl, V132, MV140 or MV140/V132. On day 7, after 24 h restimulation with LPS, IL-6, TNFα and IL-10 concentration in the supernatants were measured (n = 10 donors of four independent experiments). Values are mean ± SEM. Statistical significance was determined using One-way ANOVA. *P < 0.05, **P < 0.01, ***P < 0.001, and ****P < 0.0001.

To confirm that V132 induces trained in purified human monocytes, the same *in vitro* trained immunity protocol described above was performed using human monocytes isolated from healthy donors (**Figure 3A**). Initial exposure of monocytes to V132 alone did not induce pro-inflammatory cytokines, in contrast to the IL-6, IL-1β and TNFα production when stimulated with MV140 or MV140/V132 (**Figure 3B**). Cell viability was not affected in these cultures (**Supplementary Figure 1B**). Interestingly, monocytes simultaneously activated with MV140/V132 displayed significantly higher levels of TNFα and lower IL-1β than MV140 alone (**Figure 3B**). Stimulation of monocytes with the indicated conditions increased anti-inflammatory cytokine IL-1RA production, which was significantly higher upon V132 treatment than MV140 or MV140/V132 (**Figure 3B**), without IL-10 production (data not shown). The IL-1β/IL-1RA ratio was significantly higher when monocytes were treated with MV140 or MV140/V132 than with V132 (**Figure 3C**). MV140-treated monocytes showed a higher IL-1β/IL-1RA ratio than cells treated with MV140/V132 (**Figure 3C**). Monocytes treated with V132, MV140 or MV140/V132 significantly increased the production of lactate after 6 days of resting, reflecting an increased glycolysis metabolism (**Figure 3D**). Supporting our previous data with PBMC, monocytes trained with V132 produced higher amounts of lactate compared to the other assayed stimuli, without significant differences observed in this case (**Figure 3D**). V132-trained monocytes produced significantly higher amounts of IL-6 and TNFα upon re-stimulation 7 days after first priming and resting with LPS, without IL-10 induction (**Figure 3E**). Collectively, these data indicate that in the assayed conditions V132 but not MV140 or MV140/V132 induced trained immunity also in purified human monocytes.

### V132-trained human monocytes display enhanced innate pro-inflammatory responses upon secondary stimulation with MV140

**Figure 4:**
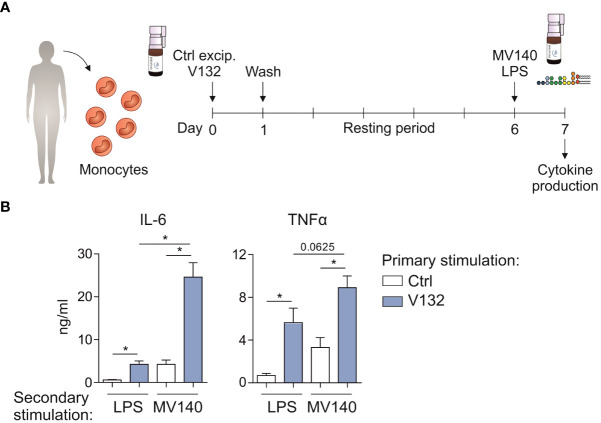
V132-trained monocytes display enhanced innate immune responses upon *in vitro* secondary treatment with MV140. **(A)** Human *in vitro* model for the induction of trained immunity in monocytes. **(B)** Monocytes were incubated for 24 h with control excipients (ctrl) or V132 (primary stimulation). On day 7, after 24 h restimulation with LPS or MV140 (secondary stimulation), IL-6 and TNFα concentration in the supernatants were measured (n = 6 donors of two independent experiments). Values are mean ± SEM. Statistical significance was determined using Wilcoxon test. *P < 0.05.

Next, we wanted to assess whether V132-trained monocytes could display an enhanced response to MV140 used as secondary stimulus (**Figure 4A**). V132-trained monocytes treated with MV140 produced significantly higher concentration of IL-6 and TNFα compared to control monocytes (**Figure 4B**). The production of pro-inflammatory cytokines by V132-trained monocytes was higher upon secondary stimulation with MV140 than LPS (**Figure 4B**). Collectively, these data indicate that exposure of monocytes to V132 significantly enhances their capacity to mount potent pro-inflammatory responses upon exposure to MV140.

### V132 induces *in vivo* trained immunity in mice and enhances Th17 responses triggered by MV140

**Figure 5:**
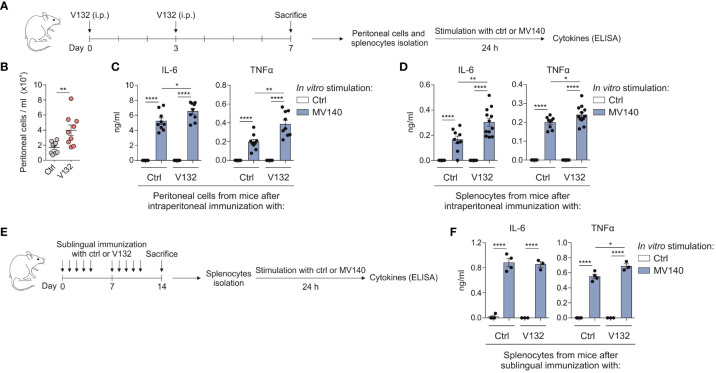
V132 induces trained immunity in mice and enhances innate immune responses after MV140 *in vitro* stimulation. **(A)** Scheme of the intraperitoneal immunization protocol and analysis of induced systemic response; i.p., intraperitoneal; ctrl; control excipients. **(B)** Concentration of peritoneal cells in the peritoneal cavity 4 days after last intraperitoneal immunization (n = 9 mice of two independent experiments). **(C)** Cytokine production by peritoneal cells from the indicated mice, stimulated *in vitro* with ctrl or MV140 for 24 h (n = 9 mice of two independent experiments). **(D)** Cytokine production by splenocytes from the indicated mice, stimulated *in vitro* with ctrl or MV140 for 24 h (n = 9-12 mice of two independent experiments). **(E)** Scheme of the sublingual immunization protocol and analysis of induced systemic response; ctrl; control excipients. **(F)** Cytokine production by splenocytes from the indicated mice, stimulated *in vitro* with ctrl or MV140 for 24 h (n = 3-4 mice of one independent experiment). Values are mean ± SEM. Statistical significance was determined using Unpaired *t* test. *P < 0.05, **P < 0.01, ****P < 0.0001.

To assess the *in vivo* relevance of V132-induced trained immunity, BALB/c mice were immunized by intraperitoneally administration of control excipients or V132 as shown in **Figure 5A**. Intraperitoneal immunization with V132 significantly increased the accumulation of infiltrating cells into the peritoneal cavity compared to controls (**Figure 5B**). Peritoneal cells and splenocytes from both V132- and control mice did respond to MV140 *in vitro* by producing IL-6 and TNFα (**Figure 5C, D**, respectively). However, cells derived from V132-immunized mice produced significantly higher levels of IL-6 and TNFα than those from control mice (**Figure 5C, D**). To further confirm the results when V132 was administered through the sublingual route, BALB/c mice were immunized as shown in **Figure 5E**. Splenocytes from both V132-immunized and control mice produced IL-6 and TNFα *in vitro* upon MV140 stimulation (**Figure 5F**). Interestingly, the production of TNFα, but not of IL-6, were higher in mice immunized sublingually with V132 (**Figure 5F**).

**Figure 6:**
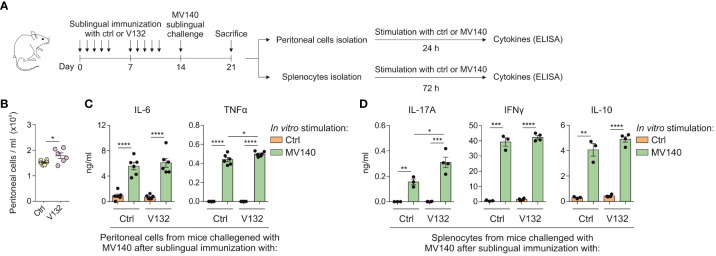
V132 induces trained immunity in mice and enhances adaptive immune responses against MV140. **(A)** Scheme of the sublingual immunization protocol and analysis of induced systemic response; ctrl; control excipients. **(B)** Concentration of peritoneal cells in the peritoneal cavity 7 days after stimulation with MV140 (n = 6 mice of one independent experiment). **(C)** Cytokine production by peritoneal cells from the indicated mice, stimulated *in vitro* with ctrl or MV140 for 24 h (n = 6 mice of one independent experiments). **(D)** Cytokine production by splenocytes from the indicated mice, stimulated *in vitro* with ctrl or MV140 for 72 h (n = 3-4 mice of one independent experiment). Values are mean ± SEM. Statistical significance was determined using Unpaired *t* test. *P < 0.05, **P < 0.01, ***P < 0.001, and ****P < 0.0001.

To assess potential effects of V132-induced training *in vivo* on adaptive immune responses, BALB/c mice immunized sublingually with control excipients or V132 were treated *in vivo* with MV140 as shown in **Figure 6A**. Sublingual immunization with V132 and subsequent sublingual treatment with MV140 significantly increased the cell infiltration into the peritoneal cavity (**Figure 6B**). Peritoneal cells from both mice groups activated *in vitro* with MV140 produced significantly higher concentrations of IL-6 and TNFα than those without MV140 stimulation (**Figure 6C**). The levels of TNFα, but not of IL-6, produced by peritoneal cells from V132-sublingually immunized mice were slightly and significantly higher than those induced by peritoneal cells from control mice after *in vitro* stimulation with MV140 (**Figure 6C**). To assess whether V132 enhances specific T cell responses to MV140, splenocytes from immunized mice (**Figure 6A**) were stimulated *in vitro* with or without MV140 (**Figure 6D**). As shown, mice both immunized sublingually with V132 or control mice produced a significant IL-17A, IFNγ and IL-10 *in vitro* response to MV140 stimulation (**Figure 6D**). Remarkably, the levels of IL-17A, but not IFNγ or IL-10, produced by splenocytes from V132-immunized mice after *in vitro* MV140 stimulation were significantly higher than those induced by splenocytes from control mice (**Figure 6D**), indicating that sublingual V132 vaccination potentiates Th17 adaptive immune responses in mice against MV140.

## Discussion

In this study, we demonstrate that V132, a heat-inactivated, whole-cell *Candida albicans* sublingual vaccine, induces trained immunity in human PBMC and purified monocytes, which in turn significantly enhances immune responses triggered by the MV140 sublingual vaccine, a whole-cell, heat-inactivated, polybacterial formulation. V132-induced trained immunity is mediated by the induction of metabolic reprogramming mirrored by increased glycolysis and lactate production as well as enhanced oxidative phosphorylation. In addition, V132 induces epigenetic reprogramming at the level of H3K27ac and H3K9me3 within the promoter regions of *IL6* and *TNFA* genes, which results in enhanced transcription of these pro-inflammatory genes upon subsequent exposition to MV140. Supporting these findings, intraperitoneal or sublingual *in vivo* immunization of mice with V132 generates peritoneal cells and splenocytes able to display enhanced innate immune responses upon *in vitro* stimulation with MV140. Remarkably, splenocytes from V132-sublingually immunized mice and treated *in vivo* with MV140 show a significant increase of Th17 responses as detected by *in vitro* stimulation with MV140. Overall, we shed light into unprecedented molecular mechanisms by which V132 might contribute to enhance innate and adaptive immune responses triggered by MV140, which might help to pave the way for future novel interventions for GUTIs.

GUTIs, including RUTIs and RVVCs, are among the most prevalent bacterial and fungal infections worldwide, requiring repeated administrations of antibiotics and antifungals (2, 47). Long-term use of antibiotics and antifungals are associated with the development of drug resistances and microbiota alterations. Alternative strategies able to overcome such deleterious effects while preserving efficacy are highly needed (1, 13). In this regard, different approaches have been considered for RUTIs (21, 48, 49) and RVVCs (50–52). Among these alternatives, several studies have focused on the development of TIbV over the last years (19, 24, 53). TIbV induce innate immune memory, thus conferring heterologous protection against a broad range of pathogens (19). The use of TIbV might also offer great potential for infections without or with no effective treatments available, for pathogens with high mutation rates as well as for diseases in which co-infections takes place such as GUTIs (19). The sublingual formulation MV140 prevents RUTIs in up to 80-90% of patients (34–41). RUTIs are also frequently associated with RVVCs, with the V132 *C. albicans* formulation being developed as a potential alternative to antifungals (22). We previously showed that the use of MV140/V132 in a single sublingual preparation enhanced IgG and IgA responses in mice against all the antigens contained in the vaccine preparation (22). In addition, MV140/V132 imprinted human DCs with the capacity to prime potent adaptive immune responses against both related and unrelated antigens (22). However, whether V132 could induce trained immunity and how this could contribute to modulate the innate and adaptive immune responses induced by MV140 remained elusive until this work.

Here we show that V132-induced trained immunity in human PBMC and monocytes significantly enhanced innate and adaptive immune responses triggered by MV140. β-glucan, the cell wall component of *C. albicans* is, together with live attenuated *Mycobacterium bovis* BCG vaccine, one of the best characterized trained immunity inducers (30, 43, 44). First stimulation of monocytes with V132 did not induce production of pro-inflammatory cytokines after 24h (43), and only the inhibitory cytokine IL-1RA was observed, as previously described for other trained immunity inducers (54, 55). The binding of IL-1RA to the same receptor as IL-1β, inhibits IL-1β- induced signaling and controlling inflammation during infections (56). *In vitro*, V132 training increases IL-6 and TNFα production after second stimulation with LPS or MV140.

To confirm the relevance of our findings, mice were immunized with V132 to assess its capacity to induce trained immunity *in vivo*. V132 *in vivo* immunization significantly enhanced immune responses when peritoneal cells and splenocytes were stimulated *in vitro* with MV140. Previously, studies reported that TIbV might also potentiate adaptive responses against different pathogens (19, 20, 57–59). Our *in vivo* results showed that the sublingual immunization of V132 and a subsequent stimulation with MV140 in mice promotes specific systemic Th17 responses, suggesting the potential immunomodulatory capacity of V132 to enhance adaptive immune responses. In contrast to V132, when human monocytes were primed *in vitro* with MV140 or MV140/V132 a significant increase of concentrations of pro-inflammatory cytokines and IL-1RA was detected after 24 h. However, after 6 days of resting, IL-6 and TNFα production upon second stimulation of monocytes with LPS were very low, suggesting that MV140 or MV140/V132 generate unresponsive monocytes to the secondary LPS exposition. Supporting these results, it has been previously described that TLR signaling through LPS may counteract the induction of trained immunity by *C. albicans* cell wall component β-glucan (58) and that LPS promotes tolerance in human monocytes, keeping the cells in an unresponsive state to secondary stimulation (33, 45). It is important to keep in mind that MV140 is composed of 75% of Gram-negative bacteria, thus containing LPS that might also partially reverse the V132-induced trained immunity. The main aim of including MV140 together V132 in the vaccine formulation is to incorporate the bacterial antigens contained in MV140 preparation to induce adaptive immune responses against all the stimuli.

Metabolic and epigenetic reprogramming are hallmarks underlying trained immunity (26, 29). Trained cells use different metabolic pathways to adapt their function for the production of energy faster and more efficiently (30–32). β-glucan-trained cells displayed a high glycolysis rate and Warburg effect but decreased oxidative phosphorylation, depending on Akt/mTOR/HIF-1a pathway (30, 31). We have previously reported that V132 activates the Akt/mTOR signaling pathway in human DCs (22). Here, we showed that V132-induced trained immunity enhances not only glycolysis with a higher lactate production, but also oxygen consumption. Arts et al. reported that BCG training also induced both glycolysis and oxidative phosphorylation in human monocytes, suggesting that different metabolic pathways can be activated depending on the training stimuli (32). Metabolic rewiring directly contributes to epigenetic reprogramming of trained monocytes (27, 32). Training of monocytes promotes epigenetic changes at promoters of genes of pro-inflammatory cytokines (27). Our chromatin immunoprecipitation experiments showed that V132 training results in an increased H3K27ac and a decreased H3K9me3 at promoter regions of *IL6* and *TNFA* genes, as previously showed for other trained immunity inducers (20, 32, 43, 60, 61).

The use of *C. albicans* as trained immunity inducer is well described, as well as its cell wall component β-glucan (43). Here, we have studied the V132 (*C. albicans*) used in a sublingual vaccine under clinical development for the prevention of recurrent vulvovaginal candidiasis. This fungal infection is associated with the use of antibiotics (6), as those indicated for treating recurrent urinary tract infections, and therefore in a number of instances both types of infections coexist (4). In this context, prior vaccination with V132 as a trained immunity inducer may be useful to potentiate MV140, a sublingual vaccine indicated for the prevention of recurrent urinary tract infections (41). It should be noted that mucosal tissues are excellent targets for inducing trained immunity by whole cell microorganisms (62, 63), supporting the approach we describe here. On the other hand, vaccination through the sublingual route induces strong immune responses in local and peripheral lymphoid organs as well as in distant mucosa such as the genitourinary tract (64–66).

In summary, we provide novel insights into the mechanisms through which V132-induced trained immunity might enhance both innate and adaptive immune responses triggered by MV140. An improved understanding of the underlying mechanisms of sublingual fungal and bacterial vaccines may well contribute to develop novel therapeutic strategies for GUTIs and other immune-mediated diseases.

## Data availability statement

The raw data supporting the conclusions of this article will be made available by the authors, without undue reservation.

## Ethics statement

Buffy coats from healthy donors were obtained after written informed consent (Sanquin Blood Bank, Nijmegen, the Netherlands). Written informed consent for participation was not required for this study in accordance with the national legislation and the institutional requirements. All mice procedures included in this study were reviewed and ethically approved by Universidad Complutense de Madrid (UCM) and Comunidad Autónoma de Madrid (CAM) within the context of project SAF-2017-84978-R, (CAM:ref.10/250312.9/18).

## Author contributions

Conceived and designed the study: OP and JD-A. Performed the experiments: LM-C (human and mice experiments), AA (mice experiments), IB and ÖB (technical support for human experiments). Provided reagents: JS, MN, JD-A, and OP. Analyzed and discussed the data: LM-C, AA, IB, ÖB, JS, MN, JD-A, and OP. Wrote the paper: LM-C and OP. All the authors revised the manuscript, contributed with revisions and approved the final version of the manuscript.

## Funding

The authors laboratories are supported by grant PID2020-114396RB-I00 to OP from MINECO, Spain, by unrestricted grant from Inmunotek under an Art.83 UCM contract to OP; The Netherlands Organization for Scientific Research (VENI grant 09150161910024 and Off Road grant 04510012010022) to JD-A; ERC Advanced Grant (#833247) and a Spinoza Grant of the Netherlands Organization for Scientific Research to MGN. LM-C is a recipient of FPU predoctoral fellowships from MINECO, Spain.

## Conflict of interest

OP has received fee for lectures or participation in Advisory Boards from Allergy Therapeutics, Amgen, AstraZeneca, Diater, GSK, Pfizer, Inmunotek SL, Novartis, Sanofi Genzyme, Stallergenes and Regeneron. OP has received research grants from Inmunotek SL, Novartis SL, MINECO, MICINNIN and CAM. JS is the founder and CEO of Inmunotek SL.

The remaining authors declare that the research was conducted in the absence of any commercial or financial relationships that could be construed as a potential conflict of interest.

## Publisher’s note

All claims expressed in this article are solely those of the authors and do not necessarily represent those of their affiliated organizations, or those of the publisher, the editors and the reviewers. Any product that may be evaluated in this article, or claim that may be made by its manufacturer, is not guaranteed or endorsed by the publisher.

## Supplementary material

Supplementary Figure 1
**(A)** Cytotoxicity assay after stimulation of human PBMC with control excipients (ctrl), V132, MV140 or MV140/V132 for 24 h. Lysed cells are included as a control (n = 6 donors of two independent experiments). **(B)** Cytotoxicity assay after stimulation of human monocytes with ctrl, V132, MV140 or MV140/V132 for 24 h. Lysed cells are included as a control (n = 6 donors of two independent experiments). Values are mean ± SEM.

## Abbreviations

Akt, protein kinase B; APC, allophycocyanin; BCG*, Bacille Calmette-Guérin*; Ctrl, control excipients; DCs, dendritic cells; ECAR, extracellular acidification rate; FITC, fluorescein isothiocyanate; FTU, Formazine Turbidity Units; GUTIs, genitourinary tract infections; H3K27ac, histone 3 acetylation of lysine 27; H3K9me3, histone 3 trimethylation of lysine 9; HIF-1a, hypoxia inducible factor-1a; mTOR, mammalian target of rapamycin; OCR, oxygen consumption rate OCR; PBMC, peripheral blood mononuclear cells; PE, phycoerythrin; PerCP, peridinin-chlorophyll-protein; RUTIs, recurrent urinary tract infections; RVVCs, recurrent vulvovaginal candidiasis; TIbV, trained immunity-based vaccines; Treg, regulatory T; UTIs, urinary tract infections; VVCs, vulvovaginal candidiasis.
