# Supplementary figures and images for: Candida albicans V132 induces trained immunity and enhances the responses triggered by the polybacterial vaccine MV140 for genitourinary tract infections
Source: Front Immunol. 2022 Nov 24;13:1066383. doi: 10.3389/fimmu.2022.1066383 (PMC9729253; doi:10.3389/fimmu.2022.1066383)

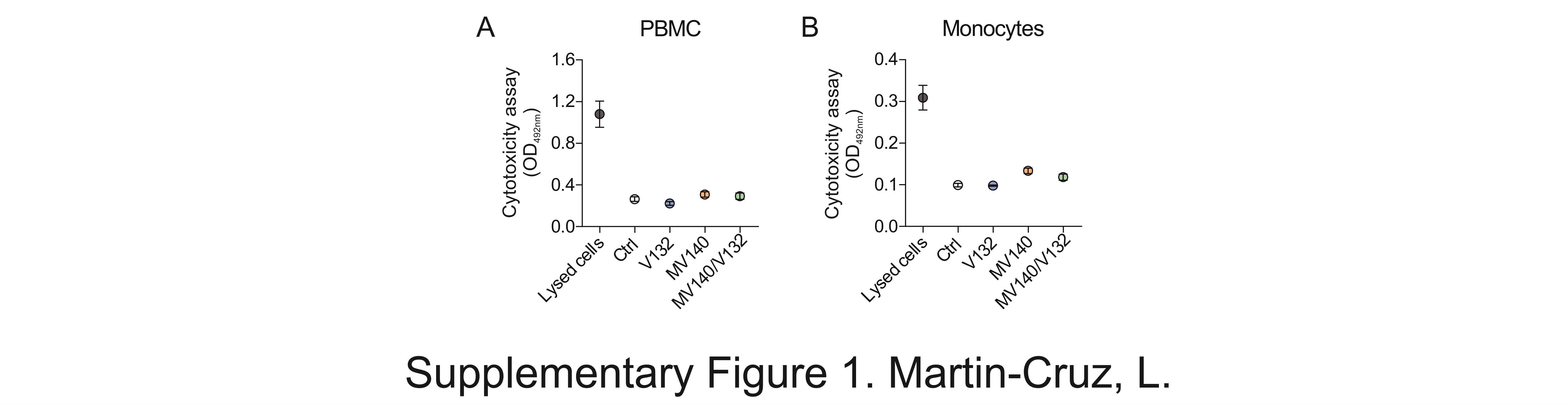

Supplement: Supplementary Figure 1 — (A) Cytotoxicity assay after stimulation of human PBMC with control excipients (ctrl), V132, MV140 or MV140/V132 for 24 h. Lysed cells are included as a control (n = 6 donors of two independent experiments). (B) Cytotoxicity assay after stimulation of human monocytes with ctrl, V132, MV140 or MV140/V132 for 24 h. Lysed cells are included as a control (n = 6 donors of two independent experiments). Values are mean ± SEM. [file Image_1.tif]
